# Clinical trials on rare diseases in Austria 2011–2021: a study using the orphanet database

**DOI:** 10.1186/s13023-026-04673-z

**Published:** 2026-10-30

**Authors:** Ursula Unterberger, Julie Bruyère-Zrelli, Trevor Lucas, Till Voigtländer, Ana Rath

**Affiliations:** 1 Center for Anatomy and Cell Biology, Medical University of Vienna, Vienna, Austria; 2 Center for Rare Diseases, University Hospital Würzburg, Würzburg, Germany; 3 INSERM, US14 ‑ Orphanet, Plateforme Maladies Rares, Paris, 75014 France; 4 Division of Neuropathology and Neurochemistry, Department of Neurology, Medical University of Vienna, Vienna, Austria

**Keywords:** Orphan medicines, Orphan medicinal products, Gene therapy, Advanced therapy medicinal products, ATMP, International rare diseases research consortium, IRDiRC, Clinical research, Clinical study

## Abstract

**Background:**

A majority of the more than 6,500 rare diseases known today still lack specific therapeutic options. Low numbers of patients and consequently participants in clinical studies, as well as high costs of development in relation to return on investment have hindered pharmaceutical companies in the development of rare disease therapeutics. However, regulatory incentives and a number of international initiatives are promoting the orphan medicine market and clinical research on rare diseases to grow quickly. We collected data on clinical trials and observational studies for rare diseases in Austria for the decade between 2011 and 2021 using the Orphanet database. Our aim was to explore the development of clinical research on rare diseases during this pivotal decade with prominent advances in the field, using the Austrian data as an example for a small-to-medium-sized European country with a highly developed healthcare system.

**Methods:**

We performed a systematic search in three large clinical study registers (the EU Clinical Trials Register, the ClinicalTrials.gov database of the National Institutes of Health, and the German Clinical Trials Register), entered the data in the Orphanet database and analysed them with regard to numbers, types of trials, rare diseases included, and medicinal products used.

**Results:**

Our results show that the numbers of clinical trials on rare diseases have been rising steadily, with the most prominent fields being rare cancers, followed by rare autoimmune and autoinflammatory diseases. The most notable growth rates in therapeutic development strategies were seen for monoclonal antibodies, while small molecules, including the large group of kinase inhibitors, are the most frequently used products overall. Trials using advanced therapy medicinal products were still scarce in comparison.

**Conclusions:**

Taken together, clinical research in the rare disease field is increasing profoundly. While medicinal products targeting genetic diseases are still scarce, especially monoclonal antibodies are increasingly used in clinical trials on rare diseases, in addition to small molecule drugs. A profoundly different picture of clinical research on rare diseases and of the orphan medicine landscape can be expected in the following decade.

**Supplementary Information:**

The online version contains supplementary material available at https://doi.org/10.1186/s13023-026-04673-z.

## Introduction

In the European Union (EU), a rare disease (RD) is defined by a prevalence of less than 5 persons affected in 10,000 residents [1]. More than 6,500 RDs are known to date [2], with up to 30 million patients in the EU – a conservative estimate that does not include rare cancers, infectious diseases, and intoxications [3], so that the actual numbers may be significantly higher. Approximately 72% of RDs are of genetic origin and 70% are of paediatric onset [3]. RDs encompass all medical fields, comprising not only genetic diseases, but also rare cancers, immunological diseases, and infectious diseases, among others. Despite this heterogeneity, RDs share common obstacles when it comes to the development of specific therapies, most of all low numbers of participants in clinical studies and high costs of development in relation to return on investment. Therefore, certain regulatory incentives have been established by the EU to encourage the development of new RD therapeutics [4], following the example of the United States (US), which had passed the Orphan Drug Act as early as 1983 [5]. Although the vast majority of RDs still lack specific treatment options, orphan medicinal products (OMPs) are no longer a niche and the orphan medicine market has been growing much faster than the non-orphan market [6]. The rapid growth of the field of OMPs and clinical research on RDs is partly due to an increasing number of initiatives to support diagnostics, research, and development in the RD field by overarching multinational efforts. As an outstanding example, in 2011, the International Rare Diseases Research Consortium (IRDiRC) was launched by the European Commission (EC) and the US National Institutes of Health (NIH). One of the main goals was the development of 200 new therapies by 2020 [7]. This goal was reached well in advance in early 2017, so that new, even more ambitious, goals could be set [8]. In the frame of accompanying monitoring efforts to gain an overview of possible effects of the IRDiRC initiative, the IRDiRC scientific secretariat, in collaboration with Orphanet, collected information on projects funded by IRDiRC members [7]. While the majority of projects were in basic and pre-clinical research [7], monitoring of clinical research is of particular interest, since it reflects direct benefits for RD patients. In Austria, we therefore decided to collect information on all clinical studies carried out in our country not only prospectively, but also retrospectively, beginning at the launch of IRDiRC in 2011, and including the following decade. Austria is a relatively small EU member state with approximately 9 million inhabitants and has participated in IRDiRC only indirectly via the Austrian Science Fund, which was a partner in the European Joint Programme on Rare Diseases from 2019 to 2023 and is now a partner in its follow-up programme, the European Rare Diseases Research Alliance. In clinical research, Austria typically profits from large multinational clinical trials with at least one trial site in an Austrian clinic, so it is expected that the situation in other EU countries can to some extent be extrapolated from the Austrian data. Of note, the publicly available clinical study databases do not distinguish between studies on RDs and non-rare diseases. While some do offer a filter function for RDs, this is typically unreliable. Orphanet is therefore still the only database providing specific data on RD research. On the other hand, data collection for Orphanet had to be performed manually until very recently, so that exhaustive and specific data on RD studies are still lacking, especially for previous years. We therefore performed a systematic search of the biggest public clinical study registers beginning with the year 2011 and including the following decade and subsequently entered the datasets in the Orphanet database. Thus, a practically complete collection of RD clinical studies with Austrian participation carried out between 2011 and 2021 could be analysed and was at the same time made available to the public via the Orphanet website. The aim of our investigation was to explore the development of clinical research on RDs during the decade from 2011 to 2021, which brought about prominent advances in the RD field, using the Austrian data as an example for a small-to-medium-sized European country with a highly developed healthcare system.

## Methods

Information on RD clinical studies (see Table 1, for a definition of the term) [9] with Austrian participation (i.e. at least one study site in Austria, including both national and inter-/multinational studies) was collected and entered in the Orphanet database by the team of Orphanet Austria at the Medical University of Vienna. Accordingly, the basis for including a study in our data collection was that it was on a disorder included in Orphanet where the European definition for RDs applied. In order to achieve completeness, the following clinical study registers were searched: the EU Clinical Trials Register [10], the ClinicalTrials.gov database of the NIH [11] and the German Clinical Trials Register [12]. The search was performed manually by first retrieving all studies (i.e. studies concerning both rare and non-rare diseases) listed in the respective register for Austria with a start date within the time period analysed via the public search window and then examining the inclusion criteria in search of studies on RDs, thereby applying the Orphanet eligibility criteria according to the internal standard operating procedure (SOP). The SOP describes the general process for data collection, registration in the database and quality control. Briefly, to be eligible for Orphanet entry, a study must specifically deal with a RD or a group of RDs, i.e. the RD or group of RDs must be mentioned explicitly in the inclusion criteria. Only studies with at least one study site in a country that is part of the Orphanet network, and/or studies funded by an IRDiRC member, are registered. In addition, only interventional studies using medicinal products are collected in the Orphanet clinical trial (another term for interventional study, see also Table 1) database. The categories in the Orphanet database comprise drug, protocol (see Table 1, for definitions of the terms), gene therapy, cell therapy, vaccine, and medical device trials. Trials on other types of interventions – such as surgery, diet, physical exercise, or cognitive therapy, among others – as well as observational and diagnostic studies are not included in Orphanet’s clinical trial database. Trials on other types of interventions and diagnostic studies are not recorded in Orphanet at all, while observational studies are registered separately as research projects, see below. Interventional trials on medicinal products are from here on referred to as interventional (clinical) trials (or just trials) for the sake of simplicity. Also, we are using the term ‘clinical study’ only if we are explicitly addressing interventional trials *and* observational studies. The Orphanet dataset for interventional clinical trials consists of the official title of the trial, the type of medicinal product used (drug, protocol, gene therapy, cell therapy, vaccine, medical device trial, see before) and the medicinal product itself, the RD or group of RDs concerned, the recruitment status, a unique identifier of the trial used in one of the trial registers, the phase of the trial, the geographical coverage (national, European or international beyond Europe), the start and end date, the primary sponsor and/or funding body, information on whether the trial is multicentric or not and is funded by an IRDiRC member or not, and a url for the trial protocol. Of note, we did not include the name of the principal investigator(s) of a trial in our data collection but entered only the country code AT and the address of the Austrian trial site(s) as a location.

**Table 1:** Terminology

Clinical study	‘A research study involving human volunteers (also called participants) that is intended to add to medical knowledge. There are two types of clinical studies: interventional studies (also called clinical trials) and observational studies’*
Clinical trial	‘Another name for an interventional study’*, also interventional (clinical) trial
Observational study	‘A type of clinical study in which participants are identified as belonging to study groups and are assessed for biomedical or health outcomes. Participants may receive diagnostic, therapeutic, or other types of interventions, but the investigator does not assign participants to a specific intervention/treatment’*
Drug clinical trial	‘A clinical trial evaluating a new substance in association with other established protocols is considered a drug clinical trial’**
Protocol clinical trial	A ‘trial which tests alternative protocols for medicines that already meet the standards of safety, quality and efficacy, and are granted a marketing authorization’** An example are new chemotherapy regimens of already approved medicines.

* According to the ClinicalTrials.gov glossary; ** According to the Orphanet Standard Operating Procedure

As mentioned before, observational clinical studies are registered separately in Orphanet as research projects flagged ’observational clinical study’. All studies fulfilling the definition for the term ‘observational study’ according to the ClinicalTrials.gov glossary (see Table 1, for a definition of the term) [9] are included. Here, the dataset comprises only the official title, the RD or group of RDs concerned, the medicinal product studied (if applicable), the start and end date, the funding body, information on whether the study is multicentric or not and whether it is funded by an IRDiRC member or not, and a url or abstract for a description. Again, names of principal investigators were not entered and only the country code and address(es) were stated.

Data on studies with Austrian participation and a start date between 01/01/2011 and 12/31/2021 were then retrieved from the Orphanet database in TXT format, using the internal PLATOR software (date of extraction 04/18/2025). Importantly, results may vary slightly depending on the extraction date, due to the continuous evolution of the Orphanet classification. It should be noted at this point that a seamless collection of fully curated datasets in Orphanet of Austrian clinical studies was not available beyond 2021, and the 10-year time span from 2011 to 2021 seemed like a reasonable basis for this paper.

In addition to the evaluation of information derived directly from the Orphanet database, some analyses regarding medicinal products used and groups of RDs included were performed manually, based on newly introduced categories that did/do not exist as such in Orphanet. Specifically, ‘human/animal tissue/organ’ was added as an extra subcategory to the medicinal products used in interventional clinical trials. Drugs were further categorised by pharmacological class/mechanism of action or chemical/biological structure. Similarly, gene therapies were sorted according to nature and functionality. RDs were categorised as rare autoimmune/autoinflammatory diseases, genetic diseases, neoplasms and ‘other’, and rare neoplasms as haematological neoplasms, rare carcinomas, nervous system neoplasms, sarcomas and ‘other’.

**Fig. 1:**
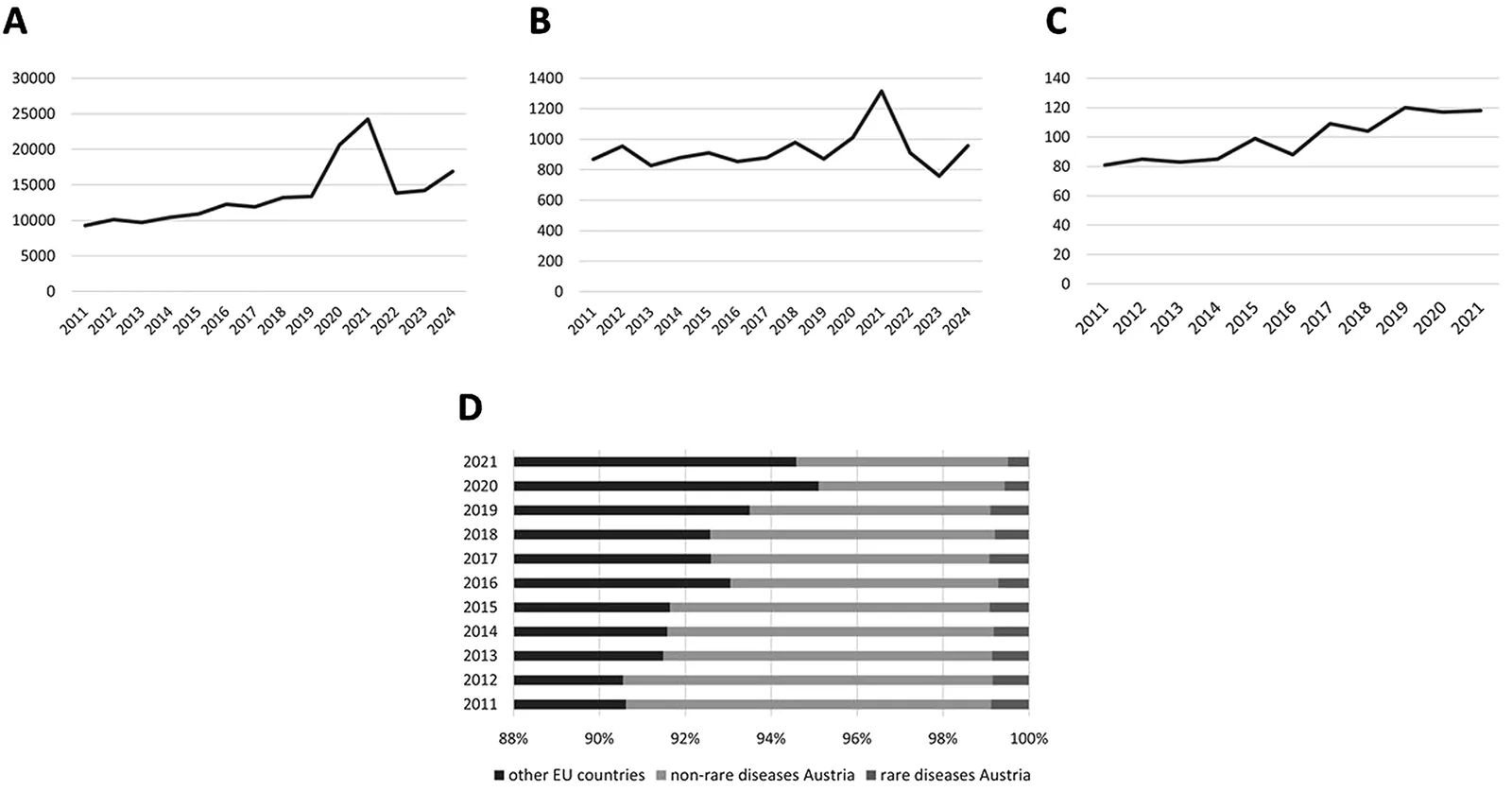
Clinical studies on rare and non-rare diseases in Austria and the EU. **(A)** Clinical studies (rare and non-rare diseases, observational and interventional) in all EU member states (including Austria) from 2011 to 2024 (Spearman’s Rho (ρ) = 0.9253, *p* < 0.001). **(B)** Clinical studies (rare and non-rare diseases, observational and interventional) in Austria from 2011 to 2024 (no significant increase (p = n.s.)). **(C)** Clinical studies (interventional and observational) on RDs in Austria from 2011 to 2021 (ρ = 0.9385, *p* < 0.05). Y-values in (**A**) to (**C**) represent absolute numbers of both interventional and observational studies. **(D)** Numbers of clinical studies (rare and non-rare diseases, observational and interventional) in EU member states excluding Austria, numbers of studies (observational and interventional) on non-rare diseases in Austria, and numbers of studies (observational and interventional) on RD in Austria are shown as fractions of 100%

Data extraction from the WHO’s International Clinical Trials Registry Platform ICTRP [13] was performed on 07/14/2025 (data in Fig. 1) and 02/19/2026 (data in Additional file 8) using the advanced search function of the search portal. The number of all studies (rare and non-rare diseases, interventional and observational) started in the EU each year between 2011 and 2024 was obtained by including all 27 EU countries in the search. The same search was then performed only for Austria. Of note, figures for 2022–2024 were included although we did not have these data for RDs in order to demonstrate that the 2020/21 peak in studies was limited to the time of the pandemic.

Statististical analysis: Spearman rank correlations were calculated for the different datasets using the Statgraphics Centurion 19^®^ software. Correlation is considered weak / moderate / strong at Spearman’s rank correlation coefficients (Rho, ρ) of ± 0.10 to 0.29 / ±0.30 to 0.49 / ±0.50 to 1.00. p-values of < 0.05/0.01/0.001 are considered significant / highly significant / very highly significant. Results of the statistical analyses are stated in the figure legends and in the original datafiles.

## Results

### Interventional and observational clinical studies on rare and non-rare diseases in Austria and the EU

Overall numbers of clinical studies in Austria and the EU obtained from ICTRP (all diseases) and Orphanet (RDs) databases are shown in Fig. 1. In general, studies were assigned to a certain year according to international start date (information on when a study started in Austria was not available). Of note, numbers of studies obtained from ICTRP include presumably very small numbers of trials on other types of interventions than medicinal products, as well as diagnostic studies. Figure 1A shows a significant increase in the number of all studies (rare and non-rare diseases, interventional and observational) in the EU between 2011 (9,260 studies) and 2024 (16,873 studies). In contrast, only studies on RDs increased accordingly in Austria (Fig. 1C and D; from 81 studies in 2011 to 118 in 2021 (data available only until 2021)), while the overall number of studies in Austria (from 869 studies in 2011 to 956 in 2024), as well as the number of studies on non-rare diseases did not grow proportionally as compared to the EU (Fig. 1B and D). Of note, the total number of studies peaked during the pandemic in Austria and the EU, while studies on RDs did not peak during this time at least in Austria (European data on RD studies not available).

**Fig. 2:**
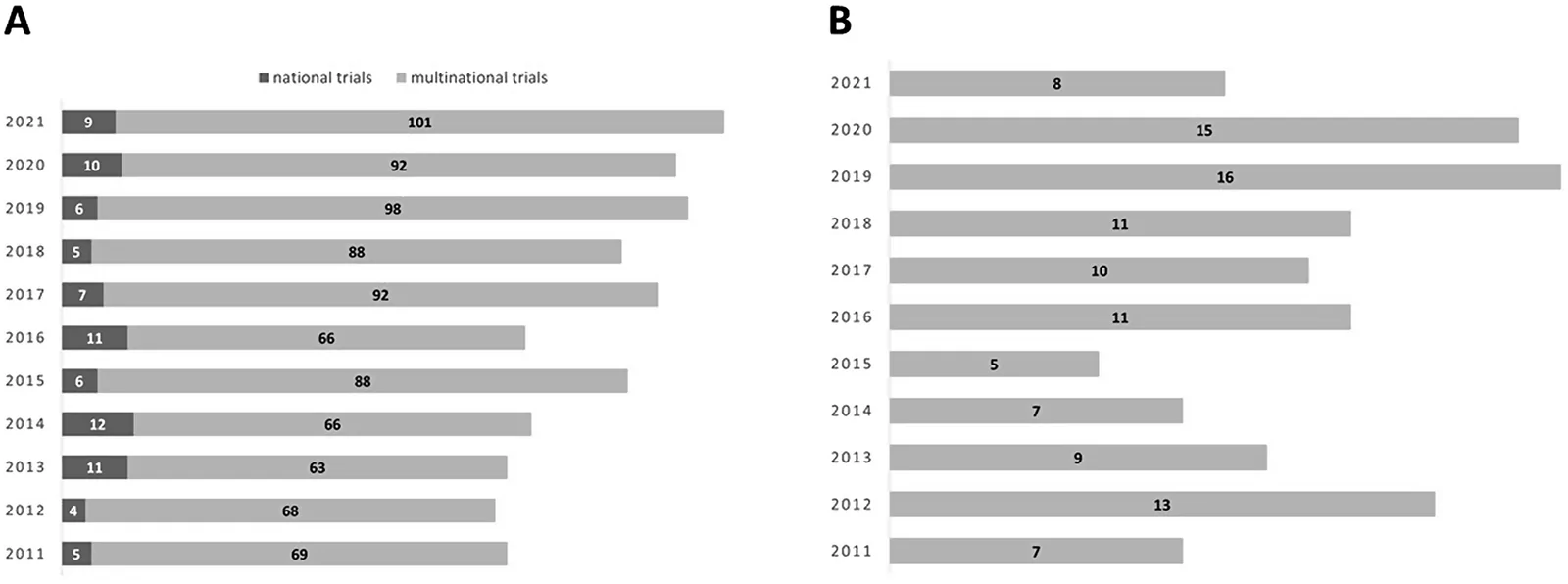
Interventional and observational clinical studies on RDs in Austria from 2011 to 2021. X-values represent absolute numbers of studies in both graphs. **(A)** Interventional trials on medicinal products are further differentiated into national (p = n.s.) and multinational (Spearman’s ρ = 0.7781, *p* < 0.05) trials (for both types of trials combined, ρ = 0.9112, *p* < 0.01). Observational studies (both national and multinational) are shown in **(B)** (p = n.s.)

With regard to studies on RDs, in total 977 interventional clinical trials with Austrian participation and a start date between 01/01/2011 and 12/31/2021 were found (Fig. 2A). Of these, 106 (10.9%) were funded by a member of the IRDiRC consortium. There was no prominent variability regarding the percentage of IRDiRC-funded trials over the years (data not shown). Similarly, 112 observational studies dealing with a RD or a group of RDs were found (Fig. 2B) and 17 (15.2%) were funded by an IRDiRC member (data not shown). Overall, the number of interventional trials on RDs conducted annually in Austria increased significantly from 2011 (74 trials) to 2021 (110 trials) (Fig. 2A). For observational studies, however, no increase was observed (Fig. 2B). For this reason, and because the overall numbers seemed too low to deduce any representative figures, observational studies were not included in any further evaluations. Of the interventional trials, the vast majority were multinational trials with Austrian participation, whereas the number of national trials was generally low (Fig. 2A; from 5 trials in 2011 to 9 in 2021, no significant increase of this type of trial was seen over the investigated time span). Comparable data were not available for other European countries.

### RDs included in interventional clinical trials in Austria between 2011 and 2021

**Fig. 3:**
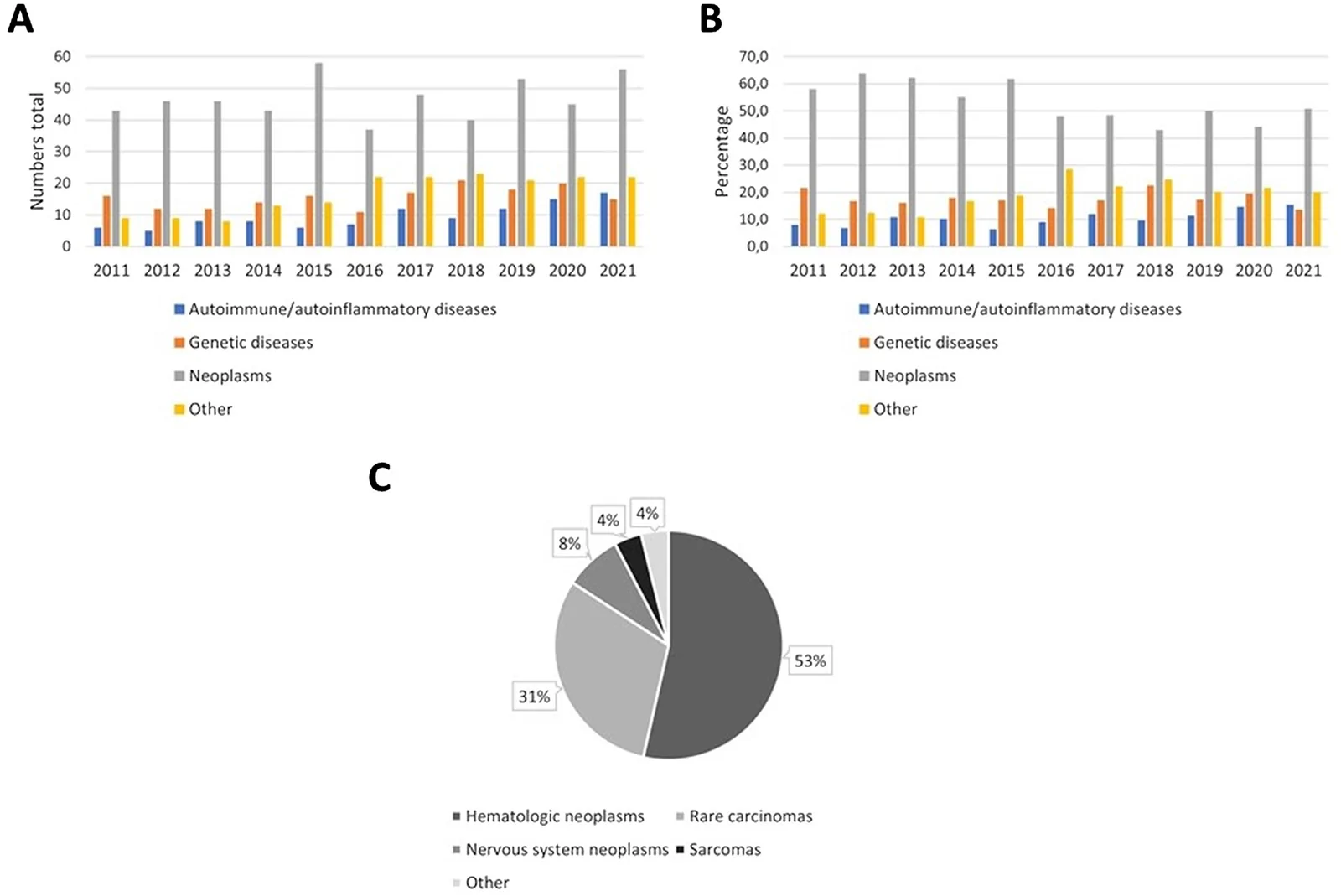
RDs included in interventional trials in Austria between 2011 and 2021.Trials on RDs carried out in Austria from 2011 to 2021 were categorised as rare autoimmune/autoinflammatory, genetic, neoplastic, and other RDs, and are shown in absolute numbers **(A)** and as percentages of all trials on RDs in Austria in the respective year **(B)** (autoimmune/autoinflammatory RDs: Spearman’s ρ = 0.8833, *p* < 0.01 (absolute numbers) / ρ = 0.7364, *p* < 0.05 (percentages); genetic RDs: p = n.s.; neoplastic RDs: p = n.s. (absolute numbers), ρ = -0.7091, *p* < 0.05 (percentages); other RDs: ρ = 0.8068, *p* < 0.05 (absolute numbers) / ρ = 0.6727, *p* < 0.05 (percentages)). **(C)** Pie chart showing trials on haematological neoplasms / rare carcinomas / nervous system neoplasms / sarcomas as mean percentages of all trials on rare neoplasms carried out between 2011 and 2021 in Austria (percentages did not vary significantly over the years; absolute numbers of all rare neoplasms per year are available as Additional file 3). Data for the categories ‘other’ and ‘genetic diseases’ are shown in Additional files 1 (‘other’) and 2 (‘genetic diseases’)

In the present study, RDs were categorised according to four main groups, comprising rare sporadic autoimmune/autoinflammatory diseases, rare genetic (monogenic) diseases, rare neoplastic diseases and ‘other’. The number of trials conducted for these disease groups in each year are shown in Figs. 3A (absolute numbers) and 3B (percentage of all interventional trials on RDs conducted in Austria in the respective year). Overall, oncology trials were the largest category by far. Although the absolute numbers were stable over the period (from 43 in 2011 to 56 in 2021), the percentage decreased slightly but significantly (58.1% of all RD trials in 2011 to 50.9% of all RD trials in 2021). In contrast, especially trials concerning rare autoimmune/autoinflammatory diseases (the most commonly investigated disease in this group being systemic lupus erythematosus, data not shown) increased significantly over time in absolute numbers (from 6 in 2011 to 17 in 2021) as well as a percentage (from 8.1 in 2011 to 15.5 in 2021). A significant increase was also found for the group ‘other’ (absolute numbers: 9 trials in 2011 to 22 trials in 2021; percentages of all RD trials of the same year: 12.2% in 2011 to 20.0% in 2021), in which the most commonly included diseases were pulmonary arterial hypertension/chronic thromboembolic pulmonary hypertension, infectious diseases (most notably the tick-bite related entities tick-borne encephalitis and Lyme disease), graft-versus-host disease and other complications of organ/stem cell transplantation (concrete numbers for the most common entities in this group, including significances, are shown in Additional file 1). No significant changes were observed for genetic diseases. Among these, the most frequently involved were rare haematological disorders (mainly the different types of haemophilia, but also Von Willebrand disease and sickle cell anaemia) and cystic fibrosis (numbers are shown in Additional file 2). With regard to rare neoplastic disorders, the largest group in this category by far were haematological neoplasms at 53% on average over the whole time span, followed by rare carcinomas (31%), nervous system neoplasms (8%), sarcomas (4%) and ‘others’ (4%) (Fig. 3C; absolute numbers underlying the pie chart are shown in Additional file 3).

### Medicinal products used in interventional clinical trials on RDs in Austria between 2011 and 2021

**Fig. 4:**
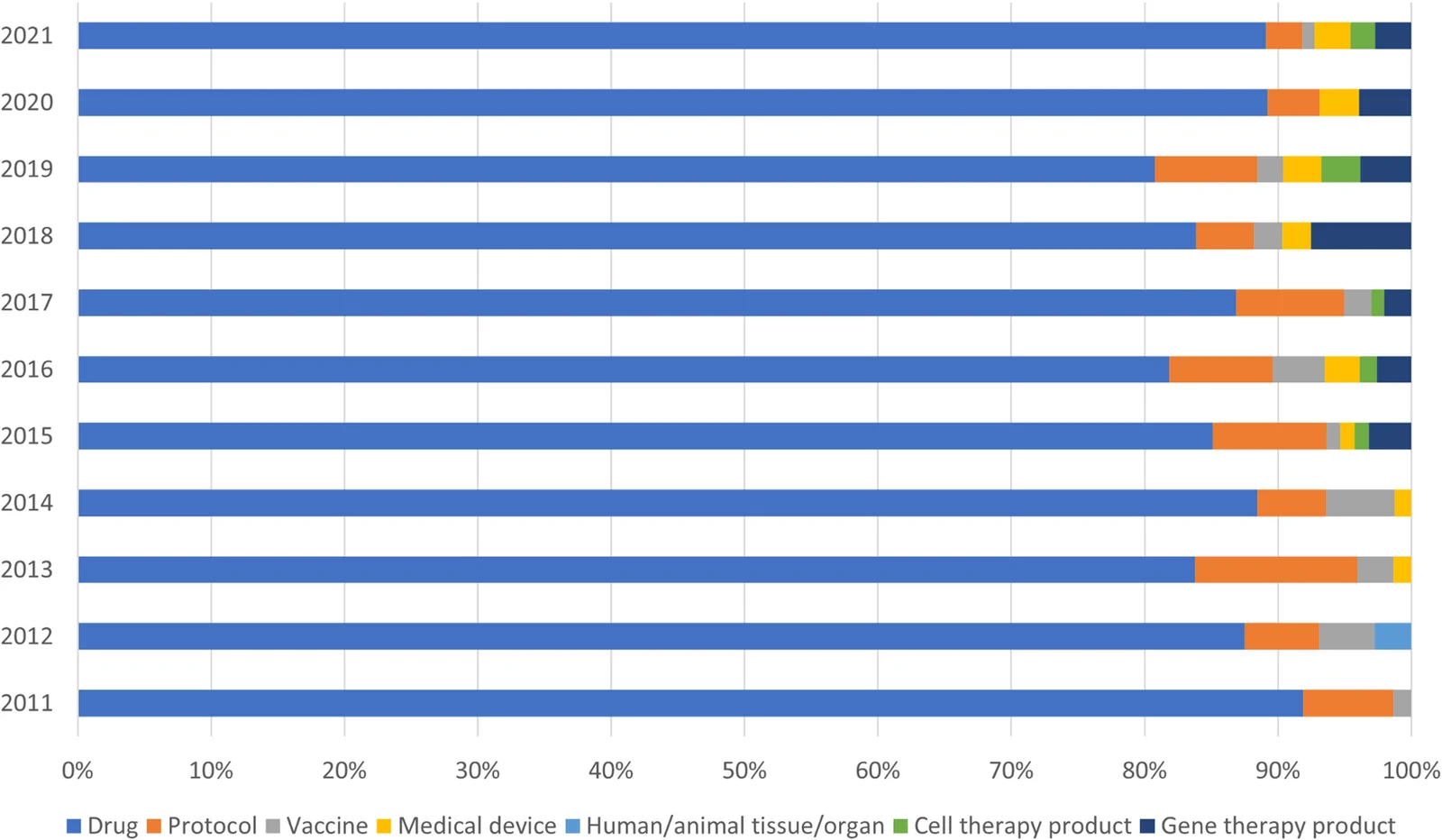
Medicinal products used in interventional trials on RDs in Austria between 2011 and 2021. Drug / protocol / vaccine / medical device / human/animal tissue/organ / cell therapy / gene therapy trials are shown as percentages of all interventional trials on medicinal products and RDs in Austria carried out during the respective year. To view the absolute numbers underlying this graph (including significances) please refer to Additional file 4

**Fig. 5:**
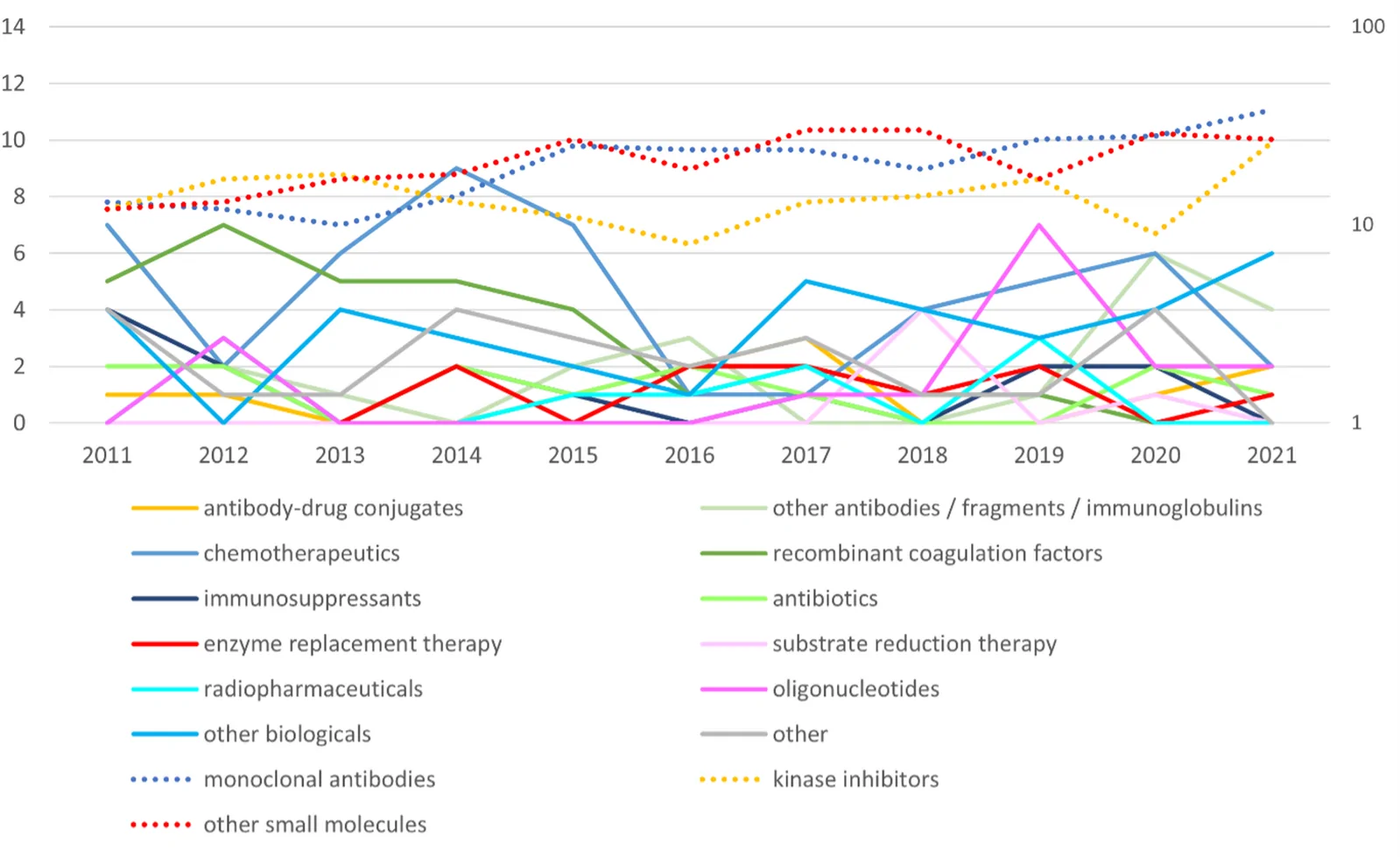
Drugs used in interventional clinical trials on RDs in Austria between 2011 and 2021. Absolute numbers of trials in which the different drugs were used are shown on the y-axes. For better discernability, monoclonal antibodies, kinase inhibitors and other small molecules (dotted lines) are shown on a secondary y-axis with logarithmic scaling on the right-hand side of the graph. Values for all other drugs are shown in linear scaling on the primary y-axis on the left-hand side. Spearman’s rank correlations were as follows: monoclonal antibodies: ρ = 0.8793, *p* < 0.01; other small molecules: ρ = 0.6819, *p* < 0.05; recombinant coagulation factors: ρ = -0.9422, *p* < 0.01; all other products did not show significant changes over the years. A graph based on relative numbers (trials for the different drugs shown as percentages of all drug trials in a respective year) is shown in Additional file 6

Categories of interventional clinical trials on medicinal products performed in Austria as obtained from the Orphanet database included drug, protocol, vaccine, medical device, cell therapy and gene therapy trials (Fig. 4 (absolute numbers underlying the graph are shown in Additional file 4); trials using human/animal tissues/organs were introduced as an additional category when evaluating the data). The vast majority were drug trials, representing more than 80% in all years, while numbers of trials in all other categories, including advanced therapy medicinal products (ATMPs, comprising gene therapy, somatic cell therapy and tissue engineered medicines) [14] were generally low, even after the first cell therapy and gene therapy trials in which Austria participated had been documented in 2015. All ATMPs together added up to 35 trials over the whole time span. CAR-T cell therapy products, which are classified as gene therapy medicinal products (GTMPs) under Regulation (EC) No 1394/2007 in the EU [14, 15], were the most commonly employed gene therapies (employed in 16 trials starting between 2015 and 2021; for a list of the specific products under investigation refer to Additional file 5), followed by Adeno-associated virus (AAV) gene therapy (5 trials), and autologous cultured epidermal grafts containing genetically modified epidermal stem cells and oncolytic virus therapy (used in 2 trials each; see Table 2), so that in total, 25 gene therapy trials were found. The different kinds of drugs used in drug trials are shown in absolute numbers in Fig. 5 (alternatively, relative numbers are shown in Additional file 6). These included monoclonal antibodies, antibody-drug conjugates, other antibodies or antibody fragments and immunoglobulins, kinase inhibitors, chemotherapeutics, recombinant coagulation factors, immunosuppressants, antibiotics, enzyme replacement therapies, substrate reduction therapies, radiopharmaceuticals, oligonucleotides, other biologicals, other small molecules and ‘others’. Of note, if more than one drug was used in a trial, all the different types of drugs were counted separately. The most prominent result was a significant increase in the use of monoclonal antibodies (from 13 trials or 19.1% of all drug trials in 2011 to 38 trials or 38.8% of all drug trials in 2021), although in general, trials using small molecules and other chemically synthesised substances outnumbered those using biologicals by far. The most frequently employed small molecules were kinase inhibitors (although no significant increase in absolute as well as relative numbers was observed between 2011 and 2021), while all other drugs were used in no more than five to ten trials per year. All numbers, correlations and significances are available from the original datafile in the Zenodo repository (see section on availability of data).

**Table 2:** Types of gene therapy products used in Austrian clinical trials on RDs between 2015-2021

CAR-T cell therapy products	16
Adeno-associated virus (AAV) gene therapy	5
Autologous cultured epidermal grafts containing genetically modified epidermal stem cells	2
Oncolytic virus therapy	2

Figures in the right column represent absolute numbers of trials in which the respective gene therapy product was used. A differentiation of the numbers per year is available from the datafile in the Zenodo repository (see section on availability of data)

### Phases of interventional clinical trials on RDs in Austria between 2011 and 2021

**Fig. 6:**
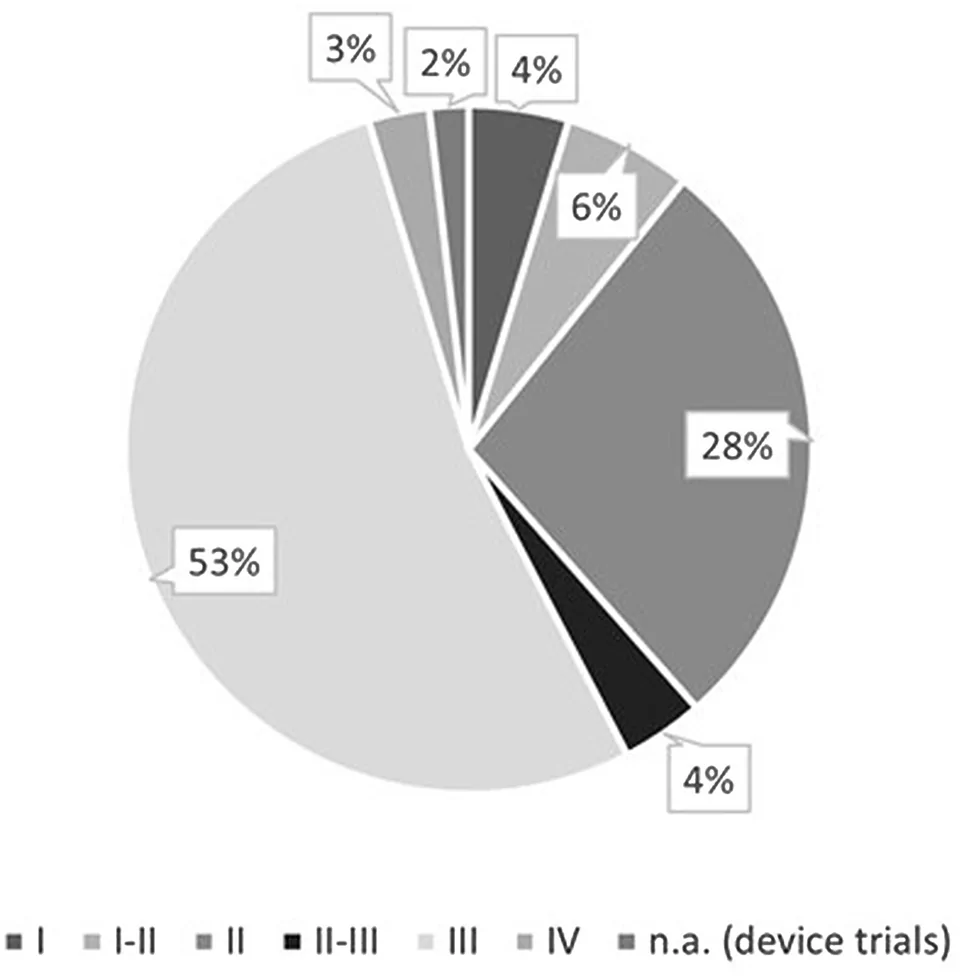
Phases of interventional clinical trials on RDs in Austria between 2011 and 2021. Pie chart showing trials in phases 1 / 1–2 / 2 / 2–3 / 3 / 4, as well as medical device trials (phase not applicable, n.a.), as percentages of all interventional trials on medicinal products and RDs carried out between 2011 and 2021 in Austria. Absolute numbers underlying the chart are available as Additional file 7

The last feature investigated were phases of clinical trials, the most frequent by far being phase 3, followed by 2, 1 and 2–3, 4 and 1–2 (Fig. 6; absolute numbers underlying the pie chart are shown in Additional file 7). Two percent were medical device trials for which no phase was stated.

## Discussion

We describe here a constant upward trend in the number of clinical studies on RDs with Austrian participation from 2011, the year of the launch of the IRDiRC consortium, and during the subsequent decade. This corroborates the findings of other studies investigating roughly the same period of time in other territories [16–21]. Interestingly, this is in contrast to the general situation of clinical studies in Austria (i.e. RD and non-RD studies taken together), which shows relatively stable numbers of studies over this period. However, it mirrors the development of clinical studies in the EU in general, where an equally steady increase can be observed. Of note, comparable information on the number of RD clinical studies was not available for the EU, or for other EU countries, so it is unknown to what extent studies on rare versus non-rare diseases are responsible for the increase on the EU level. On a side note, the total number of studies peaked during the first two years of the Covid-19 pandemic in Austria, as well as in the EU, which is in line with observations by other authors [22], but was not paralleled by a notable increase or decrease in the number of RD studies at least in Austria. Here, from an annual perspective, RD studies did not seem to be affected by the pandemic. It remains to be shown in future investigations whether the steady increase of studies – which continued in the EU as before after the intermittent peak in 2020/2021 – will be paralleled by a further increase in RD studies in Austria, and if this trend continues beyond 2024. Overall, RD studies constituted approximately 10% of all clinical studies in Austria in the early 2010s, with a steady increase over subsequent years. In general, the finding that the vast majority of interventional trials carried out in Austria were multinational trials and more than half of the trials were in phase 3 (i.e. trials including relatively large numbers of patients and therefore conducted in several countries), indicates that our results indeed reflect to a certain degree the situation in Europe. Importantly, the total number of clinical studies in Austria was and is roughly within the range (or proportional range, respectively) of other medium- or small-to-medium-sized EU countries (see Additional file 8 for some examples).

With regard to funding of studies by IRDiRC members, it was the case in 10.9% of interventional trials and 15.2% of observational studies, with no prominent changes over the years, despite the growing number of members. This may be due to the underrepresentation of Austrian organisations in the consortium or reflect the multifactorial influences on the field with a number of different measures and initiatives supporting clinical research in RDs. However, especially with prominent pharmaceutical companies joining the consortium over the years, a broader influence would have been anticipated. With respect to incentivising regulatory influences implemented in Europe during this period [23, 24], affirming a relation between those and the increasing number of trials we observed is beyond the scope of our study. Importantly, we were not investigating trials on orphan medicinal products (meaning medicines with an orphan designation) in particular, but clinical trials on RDs, which may address also medicinal products without any regulatory status and medicinal products with European marketing authorisation for RDs without orphan designation, the latter being in fact more numerous than OMPs in Europe in general [25]. Although the European Commission, in an in-depth evaluation published in 2020, concluded that the OMP regulation has had substantial impact on research and development in the field of OMPs [26], a direct causal role has not been proven, since a number of other initiatives in the RD field (such as IRDiRC, national policies, creation of the European Reference Networks [27], among others) may also have had a role.

In the present study, we chose to categorise RDs differently from the classification linearisation normally used in Orphanet (see for example [7]). The latter is based on a mixture of anatomical regions / organs or organ systems affected and pathogenic mechanisms, whereas we found – when analysing the results – a crude definition according to disease mechanisms more useful. This also helped us to avoid the significant overlaps that we would otherwise have encountered. As expected from looking at reports by other authors [7, 17, 19, 28–30], trials on rare cancers clearly outnumbered the other disease groups, comprising at least half of all RD trials in Austria annually, although the percentage decreased somewhat over the investigated decade. In parallel, an increase in absolute and relative numbers was shown for rare autoimmune/ autoinflammatory diseases, while, importantly, rare genetic diseases were relatively stable. Since the ’other’ diseases not belonging to one of these groups were also increasing (although no prominent trends regarding certain disease groups were observed in this category), we seem to be facing a situation where genetic diseases, which are in fact the largest group within RDs in general, are addressed by the smallest number of trials, while the majority of trials performed are on one of the several hundred rare cancer entities. This is also reflected in the constantly growing numbers of trials using monoclonal antibodies and the extensive use of kinase inhibitors, which are most commonly used in rare cancers (in addition to immunological disorders). Within the rare cancer field, the scenery is dominated by haematological neoplasms, corroborating the impression that although trials on RDs are generally expanding, the increase can mainly be attributed to a certain group of diseases, while a vast number of RDs are grossly underrepresented. Even when examining the genetic diseases on which trials were performed, haematological disorders (for which most commonly recombinant coagulation factors were investigated) stood out as a group, followed by a wide margin by cystic fibrosis, showing there is no diversified inclusion of different genetic diseases. However, trials on gene therapies were only started in 2015/2016 in Austria, so that we may see a profoundly different picture in the following decade. It is important to underline in this context that it would be extremely useful to extend the data collection over the following decade, and to collect more data on other European countries in the future to have a more complete overview of trends. Artificial intelligence-supported methods, which are now being used and tested by Orphanet, might make this task much easier over the coming years, although the reliability and comprehensiveness of these methods still need to be shown. Of note, until recently, automated extraction of RD studies e.g. from the ICTRP was not successful, even with specific algorithms, so that all data collection and curation steps had to be performed manually.

Regarding the proportion of medicinal products used, our results were in line with those of a previous study using Orphanet data [7], showing that, typically, more than 80% of all RD trials in Austria were drug trials, while numbers of trials in the other categories (protocol, vaccine, medical device, cell therapy, gene therapy, human/animal tissues/organs) were generally low. However, regarding trials on ATMPs, it seemed that they became a constant among all RD trials from 2015 onwards, adding up to 35 trials over the whole time span analysed. For comparison, a 2016 investigation searching the large trial databases already found 939 ATMP trials in total since 1999, with a steady increase from 34 trials in 1999 to 150 in 2014 [31] (of note, up to nearly 85% of ATMPs may target RDs, depending on the year and region [32]). Whether ATMP trials will grow further in proportion in Austria, as observed by other authors globally [31, 33], remains to be shown in future studies.

With regard to the large group of drug trials, it seemed useful to look into the nature of the drugs in more detail. Similarly to the categorisation of diseases addressed by the clinical trials, we chose to categorise the drugs used in the identified drug trials in hindsight, based on the obtained overall results. With this in mind, we found a categorisation according to pharmacological class/mechanism of action, or to chemical/biological structure, more useful than to strictly adhere to existing systems, such as the Anatomical Therapeutic Chemical Classification of the WHO. In line with previous publications [16, 17, 20], small molecules and other chemically synthesised drugs were used in the majority of trials, while biologicals were used much less frequently. However, the steadily increasing number especially of monoclonal antibodies was particularly striking and it will be interesting to see if the trend continues over the following years and in the future. Moreover, it seems reasonable to assume that monoclonal antibodies will be used in a broader spectrum of applications (in addition to cancers and immunological disorders) in the future. As mentioned before, in order to have an overview and visualise trends, it will be important to have detailed data on more countries in the future. Even if, to a certain degree, conclusions may be drawn especially for the situation in the EU when looking at one country (as in the case of the present study), we cannot determine whether differences in results compared to other reports are due to differences between countries (such as differences in resources allocated to clinical research, number of study sites, trained medical staff, regulatory processes, among others) or to different study designs and methods, data sources, etc. In any case, we did find a number of parallels with other publications, which seem to be common denominators, such as the constant increase in RD studies during the 2010s, the overwhelming majority of trials on rare cancers, or the majority of chemically synthesised medicines as compared to biologicals. It is highly likely that RD trials will keep growing more in numbers than trials on non-rare diseases, and that the overall picture – with artificial intelligence, genetic engineering, and other technologies on the rise – will look substantially different for the following decade.

## Conclusions

Taken together, our results show that the numbers of clinical trials on RDs in Austria have been rising steadily over the last decade, the most prominent field being rare cancers, followed by rare autoimmune and autoinflammatory diseases. Gene therapies for rare genetic diseases were still a largely underserved area. Since the majority of trials in which Austria participated were multinational phase 3 studies, the results can to some extent be considered exemplary for other EU countries. However, our study represents a rare example of a comprehensive, detailed analysis on this topic, and more studies – especially also for larger countries – would be highly valuable to visualise developments and gaps in the RD field. In particular, monitoring an increasing use (or a lack thereof) of ATMPs for rare genetic diseases is of high relevance.

## Supplementary Information

Below is the link to the electronic supplementary material.

Supplementary Material 1: Additional file 1 (Excel file, .xlsx): RDs of the category ‘other’ included in interventional trials in Austria between 2011 and 2021. Trials are shown in absolute numbers (A) and as percentages of all trials in the category ‘other’ in the respective year (B). (Infectious diseases exclude infections occurring in the context of cystic fibrosis. PAH = pulmonary arterial hypertension, CTEPH = chronic thromboembolic pulmonary hypertension, GvHD/other = graft-versus-host disease and other complications of organ / stem cell transplantation, n.s. = not significant)

Supplementary Material 2: Additional file 2 (Excel file, .xlsx): Interventional trials on rare haematologic genetic diseases and cystic fibrosis in Austria between 2011 and 2021. Trials are shown in absolute numbers (A) and as percentages of all trials in the category ‘rare genetic diseases’ in the respective year (B). (n.s. = not significant)

Supplementary Material 3: Additional file 3 (Excel file, .xlsx): Absolute numbers of trials on rare neoplasms carried out between 2011 and 2021 in Austria. The table shows the absolute numbers underlying figure 3C in the main body of the article.

Supplementary Material 4: Additional file 4 (Excel file, .xlsx): Medicinal products used in interventional trials on RDs in Austria between 2011 and 2021. Numbers represent the absolute numbers of trials in which the respective type of medicinal product was used in that year (corresponding to figure 4 in the main body of the article).

Supplementary Material 5: Additional file 5 (Excel file, .xlsx): CAR-T cell products used in clinical trials on RDs in Austria between 2011 and 2021.

Supplementary Material 6: Additional file 6 (image file, .png): Drugs used in interventional clinical trials on RDs in Austria between 2011 and 2021 – relative numbers. Trials for the different drugs are shown as percentages of all drug trials in a respective year on the y-axes. For better discernability, monoclonal antibodies, kinase inhibitors and other small molecules (dotted lines) are shown on a secondary y-axis with logarithmic scaling on the right-hand side of the graph. Values for all other drugs are shown in linear scaling on the primary y-axis on the left-hand side. Spearman’s rank correlations were as follows: monoclonal antibodies: ρ = 0.7636, p<0.05; recombinant coagulation factors: ρ = -0.9613, p<0.01; all other products did not show significant changes over the years.

Supplementary Material 7: Additional file 7 (Excel file, .xlsx): Phases of interventional clinical trials on RDs in Austria between 2011 and 2021. The table shows the absolute numbers underlying figure 6 in the main body of the article (n.a. = not available).

Supplementary Material 8: Additional file 8 (Word file, .docx): Total numbers of clinical studies in Austria, Czechia and Denmark 2011 and 2019, in relation to the population of each country. Study numbers are from the ICTRP database. Numbers of inhabitants were retrieved from the Eurostat Databrowser.

## Data Availability

The datasets supporting the conclusions of this article are available in the Zenodo repository, https://doi.org/10.5281/zenodo.22705838.

## Declarations

### Author information

All authors are or were part of the Orphanet project.
