# Supplementary material for: Clinical trials on rare diseases in Austria 2011–2021: a study using the orphanet database
Source: Orphanet J Rare Dis. 2026 Oct 30;21:382. doi: 10.1186/s13023-026-04673-z (PMC13721337; doi:10.1186/s13023-026-04673-z)
Supplement: Supplementary file 1 [file 13023_2026_4673_MOESM8_ESM.docx]

| Country | Population counts 2019 (millions)^[1]^ | Clinical trials total 2011 | Clinical trials total 2019^[2]^ |
| --- | --- | --- | --- |
| Austria | 8.86 | 884 | 992 |
| Belgium | 11.46 | 1219 | 1756 |
| Czechia | 10.65 | 841 | 671 |
| Denmark | 5.81 | 900 | 1257 |

**Total numbers of clinical studies in Austria, Czechia, and Denmark 2011 and 2019, in relation to the population of each country**

1. Data from Eurostat Databrowser (<https://ec.europa.eu/eurostat/databrowser/explore/all/all_themes>, accessed 01/09/2026)
2. Numbers from 2019 were used to avoid the peaks during the pandemic in 2020/2021 (see Fig.1)
